# Neurodevelopmental Outcomes in Preterm Infants at 18 Months of Corrected Age Following Coronavirus Disease 2019 (COVID-19) Pandemic-Related Neonatal Intensive Care Unit (NICU) Care Changes

**DOI:** 10.7759/cureus.80266

**Published:** 2025-03-08

**Authors:** Nao Ohama, Shutaro Suga, Shunsuke Watanabe, Kentaro Tanaka, Koichi Kusuhara

**Affiliations:** 1 Department of Pediatrics, University of Occupational and Environmental Health, Kitakyushu, JPN

**Keywords:** cognitive development, covid-19 pandemic, covid-19 vs. language development, kangaroo mother care (kmc), neonatal intensive care unit (nicu), neurodevelopmental outcomes, parental involvement, visitation restrictions

## Abstract

Background: The coronavirus disease 2019 (COVID-19) pandemic led to significant changes in neonatal intensive care unit (NICU) protocols, including restrictions on parental visitation and suspension of kangaroo mother care. These modifications likely impacted preterm infants’ neurodevelopmental outcomes. While previous studies have examined the short-term developmental effects of the pandemic, the long-term neurodevelopmental consequences remain unclear. This study aimed to assess the impact of pandemic-related NICU care changes on the neurodevelopmental outcomes of preterm infants at 18 months corrected age in Japan.

Methodology: This retrospective, single-center study was conducted at a Japanese university hospital and included preterm infants born before and during the COVID-19 pandemic. Eligible infants were those with a gestational age of less than 32 weeks or a birth weight under 1,500 g, who subsequently underwent neurodevelopmental assessment at 18 months of corrected age using the Kyoto Scale of Psychological Development 2001 (KSPD). Infants were categorized into a prepandemic group (born before March 1, 2020) and a pandemic group (born on or after March 1, 2020). To compare demographic and clinical characteristics between the groups, the Mann-Whitney U test was employed for continuous variables and the chi-square test for categorical variables. All statistical analyses were performed using a predefined significance level of p < 0.05.

Results: A total of 44 preterm infants were included (22 per group). While there were no significant differences in birth weight or neonatal morbidities between the groups, the pandemic group had a significantly older gestational age (30 vs. 28 weeks, p = 0.04). KSPD assessments revealed that the pandemic group had significantly lower cognitive-adaptive (80 vs. 92, p = 0.01) and language-social (73 vs. 89, p = 0.04) developmental quotients (DQ) compared with the prepandemic group. Postural-motor DQ was lower in the pandemic group but did not reach statistical significance (82 vs. 98, p = 0.14). To account for potential confounders, an analysis of covariance was conducted, adjusting for gestational age, birth weight, and sex. The adjusted analysis remained consistent with the unadjusted findings, confirming significantly lower cognitive-adaptive DQ (F = 4.83, p = 0.03) and language-social DQ (F = 3.94, p = 0.04) in the pandemic group. Gestational age, birth weight, and sex were not significantly associated with any DQ scores.

Conclusions: Preterm infants born during the COVID-19 pandemic exhibited significantly lower cognitive-adaptive and language-social DQs at 18 months corrected age than prepandemic infants. These findings suggest that pandemic-related restrictions on parental involvement in the NICU may have potentially influenced neurodevelopment. Further research is needed to explore long-term developmental trajectories and interventions to support optimal outcomes in this vulnerable population.

## Introduction

Japan has one of the lowest neonatal mortality rates worldwide, at 1.0 per 1,000 live births, and boasts one of the highest survival rates for extremely low birth weight infants [1]. In neonatal intensive care units (NICUs), where neonates are separated from their families immediately after birth, family-centered care (FCC) has been implemented to facilitate early parental involvement. FCC has been shown to not only foster mother-infant bonding but also improve neonatal outcomes [2]. Kangaroo mother care (KMC), a key component of FCC, has demonstrated multiple benefits, including shorter hospital stays for low-birth-weight infants, improved weight gain, enhanced maternal milk production, and reduced maternal depression [3].

However, the coronavirus disease 2019 (COVID-19) pandemic significantly disrupted neonatal care practices worldwide, leading to stringent infection control measures that altered NICU environments. Many countries imposed restrictions on hospital visits and parental involvement, affecting the establishment of mother-infant bonding. While neonates born to severe acute respiratory syndrome coronavirus 2 (SARS-CoV-2)-positive mothers were rarely infected, NICU care practices changed considerably during the pandemic [4].

At our institution, as in many facilities globally, newborn care protocols changed substantially before and after the pandemic. Before the pandemic, NICU visitation, parental touch, bathing, direct breastfeeding, and parental feeding were permitted without restriction during visiting hours. During the pandemic, visiting hours were limited to one hour per day, and physical contact required glove use. All hospitalized neonates were managed in incubators, and bathing was restricted to predischarge procedures only. Moreover, KMC, which had been implemented following World Health Organization (WHO) standards [5], was completely suspended after the onset of the pandemic.

Given the established benefits of FCC and KMC, these restrictions likely had profound implications for neonatal development. Preterm infants, especially those born at less than 32 weeks of gestation or weighing less than 1,500 g, are at higher risk for developmental delays. Even before COVID-19, proactive monitoring of these infants’ developmental trajectories was a standard practice in many NICUs. Prior studies have reported that infants born during the pandemic exhibit developmental delays, particularly in motor and social domains at six months of age, as assessed using the Ages and Stages Questionnaire, Third Edition (ASQ-3) [6]. However, long-term developmental outcomes remain largely unexplored. In this study, we assessed neurodevelopmental outcomes at 18 months of corrected age using the Kyoto Scale of Psychological Development 2001 (KSPD), a standardized tool for evaluating cognitive-adaptive, language-social, and postural-motor development.

Given the crucial role of parental involvement in early neurodevelopment, this study aims to evaluate whether NICU restrictions during the COVID-19 pandemic affected preterm infants’ cognitive, language, and motor outcomes at 18 months of corrected age. We hypothesized that preterm infants born during the pandemic would exhibit lower neurodevelopmental scores at 18 months of corrected age due to reduced parental involvement in NICU care. Understanding these effects is crucial for developing evidence-based interventions to mitigate potential developmental delays in this vulnerable population.

## Materials and methods

Participants

This study was approved by the Ethics Committee of the University of Occupational and Environmental Health, Japan (approval no. 041205). Patients born at the Hospital of University of Occupational and Environmental Health, Japan, with a gestational age under 32 weeks or a birth weight under 1500 g were enrolled. They were evaluated using the KSPD at 18 months of corrected age.

The COVID-19 outbreak began spreading globally, including in Japan, by February 2020. On March 11, 2020, the WHO officially declared COVID-19 a pandemic. In response to the rising number of cases, our NICU implemented substantial modifications to infection control measures on March 1, 2020. These changes included restricting parental visitation to one hour per day, suspending KMC, mandating glove use for all physical contact with neonates, and limiting bathing to predischarge procedures only. Given the significant shift in institutional policies on this date, we defined the pandemic period as commencing on March 1, 2020, with the prepandemic period encompassing the time before this date.

The prepandemic group included infants born on or after September 1, 2016, who completed their developmental assessments by February 28, 2020. The pandemic group included infants born on or after March 1, 2020, who completed their assessments by May 8, 2023. The patients were divided into these two groups for a retrospective review of their medical records.

Kyoto Scale of Psychological Development 2001

To assess developmental outcomes, the KSPD was administered to eligible children who had reached the corrected age of 18 months. The KSPD, an individualized face-to-face developmental assessment extensively used in Japan, includes over 300 tests that evaluate domains such as postural-motor, cognitive-adaptive, and language-social. The assessments, conducted by trained psychologists, typically take 20-40 minutes [7]. To ensure consistency across evaluations, interrater reliability was assessed, and trained psychologists conducted all assessments following standardized protocols. During the pandemic, psychologists wore masks, but the children did not. The KSPD measures developmental age and developmental quotient (DQ), with a DQ under 70 indicating significant developmental delay, comparable to a Bayley-III cognitive score below 85 [8].

Data collection and definitions

Data were extracted from the hospital records. The gestational age at birth was defined as the number of completed weeks and days estimated by ultrasonography in the first trimester. The total length of hospital stay was defined as the number of days from birth to discharge. The discharge criteria were that the patient was at an overcorrected age of 37 weeks and that weight was >2,000 g. Chronic lung disease (CLD) was defined according to the 2001 National Institutes of Health consensus criteria as respiratory distress symptoms requiring supplemental oxygen for at least 28 days in the neonatal period, excluding congenital lung malformations [9]. Necrotizing enterocolitis was defined according to Bell et al. [10]. Intraventricular hemorrhage was classified as grades 1-4 based on ultrasound findings [11], with cranial ultrasound conducted daily during the first week of life, followed by weekly assessments thereafter. Retinopathy of prematurity was classified according to the International Classification of Retinopathy [12]. Chorioamnionitis was defined as intrapartum fever with at least two maternal or fetal inflammatory signs (e.g., tachycardia, uterine tenderness, leukocytosis, or malodorous discharge) caused by intraamniotic infection or inflammation [13]. Respiratory distress syndrome in preterm infants was defined by the presence of expiratory grunting, intercostal and subcostal retractions, and characteristic radiographic abnormalities observed shortly after birth [14]. Sepsis was diagnosed based on the acute onset of apnea, mottled skin, temperature instability, feeding difficulties, marked abdominal distension, respiratory distress, and hemodynamic instability, in conjunction with laboratory findings indicative of infection [15]. Periventricular leukomalacia was diagnosed using magnetic resonance imaging, which is considered the gold standard for detecting and quantifying hypoxic-ischemic injury in the periventricular white matter. Magnetic resonance imaging was performed for all study participants before discharge [16].

Statistical analysis

Statistical analyses were conducted using Excel Statistics version 3.20, 2019 (Social Survey Research Information Co., Ltd., Tokyo, Japan). Data were presented as the number of subjects (%) or median (interquartile range). The Mann-Whitney U and chi-square tests were used to compare the two groups, and a statistically significant difference was determined at p < 0.05.

## Results

**Figure 1:**
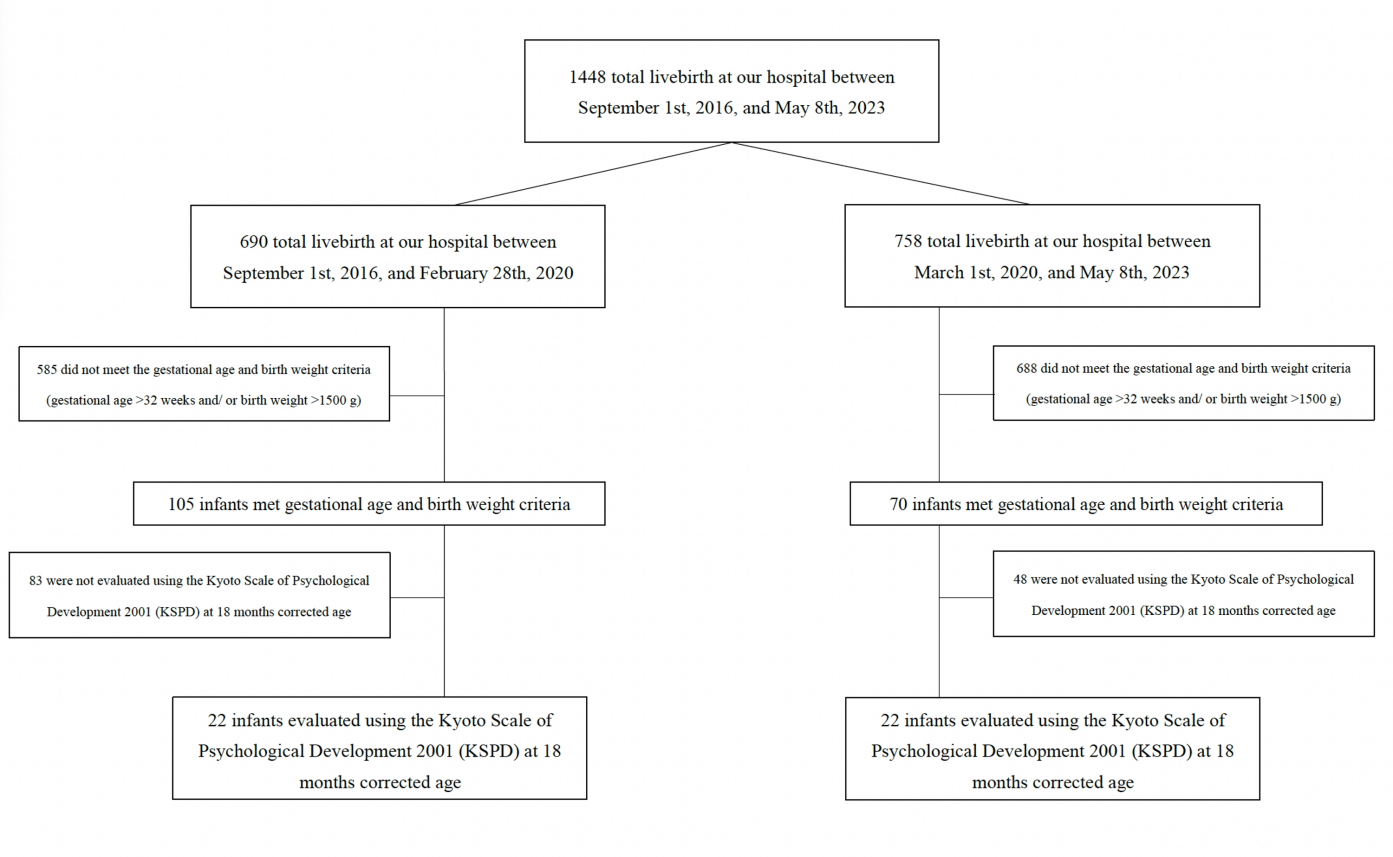
Flowchart of study participant selection based on gestational age, birth weight, and KSPD assessment KSPD: Kyoto Scale of Psychological Development 2001

Between January 1, 2019, and February 28, 2021, a total of 1,448 live births were recorded at our hospital. Of these, 690 were recorded between January 1, 2019, and February 28, 2020, while 758 occurred between March 1, 2020, and February 28, 2021. In each period, 585 and 688 infants did not meet the gestational age and birth weight criteria, respectively. Furthermore, 83 and 48 infants did not undergo the KSPD assessment at 18 months of corrected age. As a result, 22 infants from each period (total n = 44) were included as study participants (Figure 1). Their mothers (n = 44) had a median age of 32 years (interquartile range, 27-34). Of these, 25 (56.8%) were primiparous women. The prepandemic (n = 22) and pandemic (n = 22) groups were compared (Table 1). No significant differences in maternal age, where prepandemic vs. pandemic: 32 (26-34) vs. 32 (27-34) weeks, p = 0.98, or the number of primiparous women, 13 (59.1%) vs. 12 (54.5%), p = 0.77, cases of chorioamnionitis, 0 vs. 2 (9.1%), p = 0.15, administration of antenatal betamethasone, 14 (63.6%) vs. 13 (59.1%), p = 0.77, and healthcare workers, 3 (6.8%) vs. 0, p = 0.08, were found.

**Table 1:** Maternal and neonatal characteristics and outcomes of prepandemic and pandemic groups Data are presented as median (IQR) for continuous variables and as n (%) for categorical variables Group comparisons were conducted using the chi-square (χ²) test for categorical variables and the Mann-Whitney U test for continuous variables ^*^Statistical significance was defined as p < 0.05 IQR: interquartile range

Parameters	Overall (n = 44)	Prepandemic (n = 22)	Pandemic (n = 22)	Test value	p value
Total admissions during the study period	1,448	690	758	χ^2^ = 0.10	0.75
Maternal characteristics					
Age at delivery, median (IQR)	32 (27-34)	32 (26-34)	32 (27-34)	U = 243.0	0.98
Primiparous	25 (56.8%)	13 (59.1%)	12 (54.5%)	χ^2^ = 2.57	0.11
Vaginal delivery	10 (22.7%)	5 (22.7%)	5 (22.7%)	χ^2^ = 0.00	1.00
Chorioamnionitis	2 (4.5%)	0 (0%)	2 (9.1%)	χ^2^ = 0.52	0.47
Antenatal betamethasone administration	27 (61.4%)	14 (63.6%)	13 (59.1%)	χ^2^ = 0.02	0.88
Healthcare workers	3 (6.8%)	3 (13.6%)	0	χ^2^ = 2.82	0.09
Neonatal characteristics					
Gestational age, weeks	30 (27-31)	28 (27-30)	30 (28-31)	χ^2^ = 3.87	0.04^*^
Birth weight, g	1,226 (984-1,379)	1,104 (891-1,343)	1,358 (1,095-1,427)	χ^2^ = 2.21	0.13
Male sex	26 (59.1%)	12 (54.5%)	14 (63.6%)	χ^2^ = 0.09	0.76
Apgar score <4 points at 5 minutes	0	0	0	-	-
Weight gain rates during hospitalization, g/day	25 (14-35)	32 (27-44)	34 (27-46)	U = 250.5	0.85
Length of hospital stay	114 (77-213)	84 (57-112)	88 (60-116)	U = 227	0.73
Neonatal treatments					
Respiratory distress syndrome	37 (84.1%)	20 (90.9%)	17 (77.3%)	χ^2^ = 0.68	0.41
Invasive ventilation, days	9 (2-26)	9 (4-30)	11 (1-24)	U = 235.5	0.89
Supplemental oxygen, days	57 (27-87)	55 (27-92)	59 (28-84)	U = 241	0.99
Neonatal morbidities					
Chronic lung disease	32 (72.7%)	14 (63.6%)	18 (81.8%)	χ^2^ = 1.03	0.31
Necrotizing enterocolitis	0 (0%)	0 (0%)	0 (0%)	-	-
Intraventricular hemorrhage	2 (4.5%)	2 (9.1%)	0 (0%)	χ^2^ = 0.52	0.47
Sepsis	0 (0%)	0 (0%)	0 (0%)	-	-
Retinopathy of prematurity > stage 2	9 (20.5%)	5 (22.7%)	4 (18.2%)	χ^2^ = 0.09	0.76
Periventricular leukomalacia	5 (11.4%)	3 (13.6%)	2 (9.1%)	χ^2^ = 0.18	0.67

Regarding the characteristics of the neonates, the gestational age of neonates during the pandemic was significantly older: prepandemic vs. pandemic: 28 (27-30) vs. 30 (28-31) weeks, p = 0.04. Birthweight was slightly heavier during the pandemic, although this difference was not statistically significant: 1,104 (891-1,343) vs. 1,358 (1,095-1,427) g, p = 0.13. Additionally, no significant differences were observed in the sex distribution of neonates: 12 (54.5%) vs. 14 (63.6%), p = 0.54. The rate of weight gain during hospitalization was significantly faster during the pandemic: 32 (27-44) vs. 34 (27-46) g/day, p < 0.01; the length of hospital stay was slightly longer during the pandemic: 84 (57-112) vs. 88 (60-116) days, p < 0.01.

Regarding the treatment of the neonates, no significant differences were noted in invasive ventilation days: prepandemic vs. pandemic: 9 (4-30) vs. 11 (1-24) days, p = 0.87, or supplemental oxygen days: 55 (27-92) vs. 59 (28-84) days, p = 0.98. Regarding morbidities, no significant differences were found in CLD: 14 (63.6%) vs. 18 (81.8%), p = 0.18, intraventricular hemorrhage: 2 (13.3%) vs. 0, p = 0.15, or retinopathy of prematurity >stage 2: 5 (22.7%) vs. 4 (18.2%), p = 0.72.

KSPD was conducted on eligible children who were at the corrected age of 18 months (Table 2). Compared with the pandemic group, the prepandemic group had significantly higher cognitive-adaptive DQ: prepandemic vs. pandemic: 92 (88-98) vs. 80 (73-92), p = 0.01, and language-social DQ: 89 (73-103) vs. 73 (65-90), p = 0.04. Postural-motor DQ scores, 98 (80-108) vs. 82 (73-96), p = 0.14, were not significantly different between the two groups. Postural-motor DQ scores, although not showing significant differences between the groups (p = 0.14), were higher in the prepandemic group: 98 (80-108) vs. 82 (73-96). During the pandemic period, one infant was born to a mother with an SARS-CoV-2 infection, and the exclusion of this infant from the analysis did not affect the KSPD results. To further account for potential confounders, we conducted an analysis of covariance adjusting for gestational age, birth weight, and sex (Table 3). The adjusted analysis remained consistent with the unadjusted findings. The pandemic group exhibited significantly lower cognitive-adaptive DQ (F = 4.83, p = 0.03) and language-social DQ (F = 3.94, p = 0.04). In contrast, differences in postural-motor DQ (F = 0.62, p = 0.44) and overall DQ (F = 5.09, p = 0.03) followed a similar pattern. Gestational age, birth weight, and sex were not significantly associated with any DQ scores.

**Table 2:** KSPD scores in the prepandemic and pandemic groups The developmental assessments of the participants were conducted using KSPD. This evaluation tool measures an individual's overall DA and calculates the DQ using the formula: DQ = (DA/CA) × 100 Postural-motor represents the fine and gross motor functions, cognitive-adaptive indicates nonverbal reasoning and visuospatial perception assessed using materials, and language-social indicates interpersonal relationships, socialization, and verbal abilities Data are presented as median (IQR). Comparisons were performed using the Mann-Whitney U test for continuous variables ^*^Statistical significance was defined as p < 0.05 KSPD: Kyoto Scale of Psychological Development 2001; DA: developmental age; DQ: developmental quotient; CA: corrected age; IQR: interquartile range

Parameters	Overall (n = 44)	Prepandemic (n = 22)	Pandemic (n = 22)	Test value	p value
DQ in each domain					
Postural-motor	91 (76-104)	98 (80-108)	82 (73-96)	U = 179.5	0.14
Cognitive-adaptive	89 (79-97)	92 (88-98)	80 (73-92)	U = 348.0	0.01^*^
Language-social	84 (70-95)	89 (73-103)	73 (65-90)	U = 152.5	0.04^*^
Overall DQ	90 (78-96)	94 (86-99)	84 (74-93)	U = 353.5	0.01^*^
Assessment age, corrected months	20 (19-21)	20 (19-21)	20 (19-21)	U = 317.5	0.07

**Table 3:** ANCOVA results adjusting for gestational age, sex, and birth weight using standard least squares regression Sample sizes: overall (n = 44), prepandemic (n = 22), pandemic (n = 22) Postural-motor represents fine and gross motor functions, cognitive-adaptive indicates nonverbal reasoning and visuospatial perception assessed using materials, and language-social indicates interpersonal relationships, socialization, and verbal abilities ANCOVA was conducted using JMP's standard least squares regression model, with the pandemic group as a categorical factor and gestational age, birth weight, and sex as covariates ^*^Statistical significance was defined as p < 0.05 DQ: developmental quotient; ANCOVA: analysis of covariance

Dependent variable	Source	Degrees of freedom	F-statistic	p value
Postural-motor	Pandemic group	1	0.62	0.44
	Gestational age	1	0.40	0.53
	Birth weight	1	0.88	0.35
	Sex	1	0.03	0.86
Cognitive-adaptive	Pandemic group	1	4.83	0.03^*^
	Gestational age	1	1.31	0.26
	Birth weight	1	0.37	0.55
	Sex	1	0.04	0.84
Language-social	Pandemic group	1	3.94	0.04^*^
	Gestational age	1	0.24	0.63
	Birth weight	1	0.26	0.61
	Sex	1	0.23	0.84
Overall DQ	Pandemic group	1	5.09	0.03^*^
	Gestational age	1	1.14	0.29
	Birth weight	1	0.28	0.60
	Sex	1	0.18	0.67

## Discussion

This study suggests that preterm infants born during the COVID-19 pandemic may have exhibited lower cognitive-adaptive, language-social, and overall DQ on the KSPD at a corrected age of 18 months compared with those born before the pandemic. In contrast, no significant difference was observed in postural-motor DQ. These findings suggest that the potential impact of pandemic-related factors on neurodevelopment may vary across different developmental domains rather than causing a uniform delay. Our results refine previous research [6] suggesting that infants born during the COVID-19 pandemic had lower neurodevelopmental scores across multiple domains. Importantly, unlike the ASQ-3, which is based on caregiver-reported assessments, the KSPD provides an objective, standardized evaluation conducted by trained psychologists. This methodological distinction minimizes reporting bias and enhances the reliability of our findings. To account for potential confounders, we performed an analysis adjusting for gestational age, birth weight, and sex. The associations between the pandemic period and lower cognitive-adaptive and language-social development remained significant after adjustment, indicating that these differences were not solely explained by perinatal factors. In contrast, no significant difference was observed in postural-motor development, suggesting that the effects of pandemic-related factors on neurodevelopment may not have been uniform across all domains.

Although the staff composition, treatment protocols, and clinical management strategies in our NICU remained unchanged, several pandemic-related modifications in caregiving practices could have contributed to the observed developmental delays. These changes included reduced KMC, restricted visitation hours, universal masking, and the declaration of a state of emergency after discharge, which significantly altered the postnatal environment. A systematic review highlighted the significance of parental cognitive stimulation in shaping neurocognitive outcomes in preterm-born children, emphasizing its impact on language skills and broader cognitive development [17]. These findings suggest that early linguistic and cognitive interactions provided by parents play a crucial role in supporting neurodevelopmental trajectories in this vulnerable population. Given this, pandemic-related restrictions may have contributed to reduced parent-infant interactions, which could be one of several factors influencing the observed developmental differences in our cohort. Regression analysis showed that higher adult word counts in the NICU independently accounted for significant variances in cognitive and language outcomes [18], highlighting the critical role of early linguistic exposure. The severe restrictions imposed during the pandemic may have significantly reduced these crucial linguistic interactions, further exacerbating developmental deficits. Given the well-established role of parental engagement in neonatal neurodevelopment, the limited interaction opportunities imposed by infection control policies may have exacerbated the delays observed in our cohort.

In addition to NICU-specific restrictions, broader pandemic-related lifestyle modifications, including limited social interactions, reduced physical activity, and prolonged exposure to universal mask-wearing, may have contributed to developmental impairments. In early infancy, speech perception and language acquisition heavily depend on visual cues, particularly lip movement. A study demonstrated that infants who focus on a speaker's mouth exhibit enhanced speech imitation and language learning compared with those who concentrate on the speaker's eyes or other facial areas [19]. Consequently, the widespread use of face masks may have impeded early speech processing and phoneme discrimination, adversely affecting language acquisition in the pandemic group.

Beyond immediate environmental factors, the psychosocial impact of the pandemic on caregivers warrants consideration. Heightened parental stress and anxiety during the pandemic have been associated with adverse developmental outcomes in children, mediated by reduced caregiver responsiveness and alterations in the home environment [20]. While our study did not assess maternal mental health or home-based interactions, prior research suggests that increased maternal stress correlates with poorer neurodevelopmental outcomes in preterm infants. Future studies should incorporate measures of parental mental health and caregiving practices to elucidate the interplay between pandemic-related stressors and child development.

Limitations and strengths

This study has several limitations. First, as a retrospective analysis of medical records, our study could not account for postdischarge environmental variables, such as maternal education level, home stimulation, and frequency of parent-child interaction, which are known to influence developmental trajectories. Second, the relatively small sample size may have limited the statistical power to detect subtle differences in developmental outcomes. Third, as a single-center study with a relatively small sample size, our findings may not be generalizable to other NICU settings, particularly those with differing infection control policies and socioenvironmental conditions.

Despite these limitations, our study possesses notable strengths. First, the use of KSPD, an objective developmental assessment tool administered by trained psychologists, minimizes the bias inherent in parent-reported screening tools such as ASQ-3. Second, this study evaluates developmental outcomes at 18 months of corrected age, providing a longer follow-up period than previous studies that primarily assessed infants at 6-12 months. Third, the single-center design ensures consistency in treatment protocols and minimizes confounding due to variations in clinical practice, allowing for a clearer interpretation of the impact of pandemic-related changes in NICU care.

## Conclusions

This study suggests that preterm infants born during the COVID-19 pandemic may have experienced delays in cognitive-adaptive and language-social development at 18 months of corrected age. However, further research is needed to confirm these associations. These findings suggest that pandemic-related disruptions in neonatal care, parental engagement, and environmental stimulation may have long-term neurodevelopmental consequences. However, as neurodevelopmental trajectories in preterm infants may change over time, extended follow-up beyond 18 months of corrected age is necessary to assess whether these observed differences persist or if developmental catch-up occurs in later childhood. Future large-scale, multicenter studies with longitudinal follow-ups are essential to better assess the independent impact of pandemic-related factors on neurodevelopment. Additionally, further research should explore potential interventions, including enhanced parental education, early language stimulation programs, and postdischarge support strategies, to mitigate developmental delays in preterm infants born during pandemics.
